# Gentlemen’s Dress

**Published:** 1874-12

**Authors:** 


Gentlemen's Dress.—No one who thinks about it can fail to see that a very great deal of men's suffering during our summers is due to the clothes they wear. It is not the weight of the garments, or their warmth, which causes this, but their fashion. For the trouble begins with the very shirt; and surely no other garment so absurdly unfitted to the requirements of nature as one of our shirts is in summer was ever worn. It is contrived to fret us just in the place where we wish to be freest, and where we feel fretting most—the neck and wrists; and in perfect condition is just that which this fretting is surest to destroy. What incongruity could be greater than that of a sweltering man and a starched shirt? A collar made of three or four thicknesses of linen, starched stiff enough to turn off a pistol bullet, and buttoned round the throat outside a neck-band half as thick and almost as stiff; wristbands conforming to the same pattern in all respects, and a shirt-bosom like a bleached breast-plate—this is our first preparation to meet the fierce heats of June, July, and August. Then we button our trowsers tightly around our waists; we must fret our shoulders with braces, and we must put on a waistcoat.

Did any man ever reflect what a ridiculous and superfluous thing a waistcoat is in warm weather? It is the most artificial, tailor-contrived garment that the modern tailor-ridden man puts on. And in summer time what an irritating, impertinent thing it is, whether made of the softest wool or the stiffest linen. For there is another absurdity; let the waistcoat be of as light and cool a material as we can get, we must starch it. Moreover, to all this we must add the crowning absurdity of a cravat or tie of some sort around our already sextuply imprisoned necks. Truly, while men go about dressed in this style during summer, fretting themselves into real discomfort for a quarter of the year, the less they say about the absurdity of women's dresses the better—at least within hearing of the quick-witted among the quick-tongued sex.

